# Kinetics of disappearance and appearance of isoagglutinins A and B after ABO-incompatible hematopoietic stem cell transplantation

**DOI:** 10.1038/s41409-022-01737-z

**Published:** 2022-06-25

**Authors:** Baptiste Lemaire, Christophe Combescure, Yves Chalandon, Nicolas Vuilleumier, Sophie Waldvogel Abramowski

**Affiliations:** 1 Diagnostic Department, University Hospital of Geneva, Geneva, Switzerland; 2 Medicine Department, University Hospital of Geneva, Geneva, Switzerland; 3 Division of Clinical Epidemiology, University Hospital of Geneva, Geneva, Switzerland; 4 Oncology Department, University Hospital of Geneva, Geneva, Switzerland

**Keywords:** Bone marrow transplantation, Haematopoietic stem cells

## Abstract

ABO-incompatible allogeneic hematopoietic stem cell transplantation (HSCT) can be complicated by poor red cell engraftment and hemolysis, both mediated by isoagglutinins. Anecdotally, isoagglutinins indicates an activation of donor’s immunity or even relapse. Consequently, the routine monitoring of isoagglutinins could help physicians to predict the risk of complications. The purpose of this study is to investigate the time to disappearance and appearance of isoagglutinins after ABO-incompatible allogeneic HSCT. In a one-year follow-up, data of 136 ABO-incompatible hematopoietic stem cell (HSC) allogeneic transplanted patients were studied, of which 60 had major, 61 minor and 15 bidirectional incompatibility. Survival analyses were conducted and association with hematological diseases, HLA-compatibility and transplantation strategy was investigated. We observed a disappearance of isoagglutinin A in 82.0% of cases at one year with a median and 75th percentile of 38.4 and 138.6 days, respectively. For isoagglutinin B, these same values were 96.4%, 15.9 and 29.1 days, respectively. The appearance of isoagglutinin A occurred in 10.7% of cases. Disappearance of isoagglutinin A was significantly slower in patients with myeloid diseases compared to other diseases. The results of this study provide useful values to detect early risks of preventable immunohematological complications and possibly, in exceptional cases, relapse.

## Introduction

Allogeneic hematopoietic stem cell transplantation (HSCT) is indicated in many hematological diseases, both malignant (e.g. leukemia) and non-malignant (e.g. sickle cell disease). The currently used therapeutic strategies allow performing ABO-incompatible allogeneic HSCT [1]. ABO antigens are particularly important because of the severe hemolytic activity induced by their antibodies [2]. These incompatibilities are of 3 types: major (if the ABO cell-type of the donor is not compatible with the recipient, e.g. A-type donor to O-type recipient), minor (if the isoagglutinins of the donor are not compatible with the recipient, e.g. O-type donor to A-type recipient), or bidirectional (if both the previously mentioned features occur, e.g. A-type donor to B-type recipient).

Major ABO-incompatible transplantation can lead to an early hemolysis and especially an impaired red cell engraftment that could result in pure red cell aplasia (PRCA) and prolonged transfusion requirements [3–5]. Minor ABO-incompatible transplantation can also lead to hemolysis, due to donor B lymphocytes that produce antibodies to one or more of the recipient’s red cell antigens (passenger lymphocyte syndrome) [6]. However, ABO-incompatible allogeneic HSCT is as effective as ABO-compatible transplantation against oncologic disease and, according to experts, appears to have no influence on overall engraftment and survival [7–9]. But interestingly, there is a strong correlation between the presence (and titer) of incompatible isoagglutinins and the risk of post-transplantation immunohematological complications [10]. In addition, the reappearance of isoagglutinins after major ABO-incompatible allogeneic HSCT may be a sign of relapse [11]. The conditioning protocol, through its cytotoxic nature, plays a role in the persistence of isoagglutinins detected in the recipient. Among the many protocols, myeloablative conditioning should enhance isoagglutinins disappearance [12, 13]. The hematological disease, the use of anti-thymocyte globulin (ATG) as anti-rejection and GVH prophylaxis, the number of T lymphocytes contained in the graft, and the HLA mismatch might also have an impact on donor and recipient isoagglutinins [14].

Consequently, the main objective of this study is to investigate the time to disappearance and appearance of isoagglutinins A and B in patients who received ABO-incompatible allogeneic HSCT, according to different clinical factors, over a one-year follow-up.

## Materials and methods

### Study population

All patients who received an ABO-incompatible allogeneic HSCT at the Geneva University Hospital between March 2015 and December 2019 and who gave their written consent for the reuse of their data for research projects were included in this retrospective study. For each of these patients, a follow-up of the presence or absence of isoagglutinins A and B was performed over a period of one year post allogeneic HSCT. Subsequently, patients who had previously received allogeneic HSCT were excluded from the study, as were those with undetectable isoagglutinins levels at the time of transplantation.

The following immunohematological data were collected: recipient and donor blood types, date of disappearance or de novo appearance of isoagglutinins A and/or B.

The following patient data were also collected: age, gender, hematological disease, conditioning protocol, immunosuppression, HLA mismatch, HSC source, PRCA and hematological relapse.

Hematologic diseases were classified as follows: myeloid diseases (acute myeloid leukemia, mixed acute leukemia, chronic myeloid leukemia, myelodysplastic syndrome, myeloproliferative syndrome), lymphoid or lymphoproliferative diseases (chronic lymphoid leukemia, lymphoma, myeloma, acute lymphoid leukemia), others (inherited hemoglobinopathy, aplastic anemia).

Conditioning regimens were classified into two subgroups: myeloablative and reduced intensity or nonmyeloablative. Myeloablative conditioning regimen include either alkylating agents or total body irradiation in doses inducing irreversible cytopenia [15]. A list of the most used conditioning regimens and their assigned category is listed in the Appendix 1. According to ATG administration and T-cell depletion of the graft, two groups were defined. PRCA was diagnosed in case of reticulocytes count <1% and anemia after 100 days. The criteria for one-year relapse was hematological.

HLA compatibility has been defined in 2 categories: HLA-matched identical sibling (HLA 12/12 related donor) and others (HLA 10/10 unrelated donor and HLA < 10/10 related or unrelated donor).

This study has been approved by the research ethics commission of Geneva (CCER Geneva: 2020-02999).

### Biological analyses

ABO typing, including the detection of isoagglutinins (IgM) A and B, was performed every 3 to 4 days on average since the day of transplantation and until the patient was transfusion independent. The analyses were performed in the immunohematology laboratory of our institution on a Bio-Rad IH-1000 automated system with “ID-DiaClon ABO/D” gel cards (Cressier, Switzerland). A negative control is present on each gel card. Isoagglutinins were considered positive if the reaction was greater than or equal to an intensity of 0.5 (“+/−“). We did not investigate the chimerism of erythrocyte antigens because of the confounding influence of blood transfusions.

### Statistical analyses

Statistical analyses were performed using R software version 4.0.2 [16].

The risks of occurrence of four events was studied concerning isoagglutinins: disappearance of isoagglutinin A, disappearance of isoagglutinin B, appearance of isoagglutinin A and appearance of isoagglutinin B. These events were studied independently of the ABO incompatibility type of the allogeneic HSCT.

The purpose of these analyses was to study the cumulative incidence curves representing the frequency of disappearance or appearance of isoagglutinins A and B during the year after allogeneic HSCT. These curves allow us to visualize the rapidity of occurrence of the events studied, but also to estimate the median survival time, i.e. the time after which there has not yet been a disappearance or appearance for half of the participants. The 75th percentile survival time is also reported.

To account for the nature of the data, a non-parametric approach with interval survival data was used. Indeed, the exact date of disappearance and appearance of these isoagglutinins is not known. However, the data allow us to know in which time interval the disappearance or the appearance occurred. The survival package for R was used [17]. In the case where no event was observed, the survival was equal to 1.

Another objective of these analyses was to compare the risks of disappearance between isoagglutinins A and B. For this, a proportional hazard model with interval survival data was used with the icenReg package for R [18]. The hazard ratio is reported as well as the *p*-value testing for no difference between isoagglutinins A and B (null hypothesis: hazard ratio = 1). The same statistical approach was used to compare the risk of disappearance A between participants according to their subgroups in univariate and multivariate models.

In all these analyses, patients were censored at the time of death if death occurred without a disappearance or an appearance. The alpha threshold is 0.05, and all statistical tests applied are 2-sided.

## Results

### Study population

Between March 2015 and December 2019, 324 patients received allogeneic HSCT at our institution, including 137 (42.3%) ABO-incompatible patients. After exclusion of one individual with undetectable isoagglutinins, 60 (44.1%) patients received major, 61 (44.9%) minor, and 15 (11.0%) bidirectional ABO-incompatible allogeneic HSCT. The characteristics of these patients are presented in Table 1 and Table 2, and a supplement is presented in Appendix 2. Of all the patients studied, 95 (69.9%) had myeloid disease, 36 (26.5%) lymphoid or lymphoproliferative disease and 5 (3.7%) other disease. Moreover, the source of HSC was mainly peripheral blood: 48 (80.0%) for major, 52 (85.2%) for minor, and 13 (86.7%) for bidirectional ABO-incompatible allogeneic HSCT.

**Table 1:** Patient characteristics.

Characteristics	Type of ABO incompatibility		
	Major (*n* = 60)	Minor (*n* = 61)	Both (*n* = 15)
Age in years [mean (range)]	48 (0–73)	50 (1–74)	42 (1–70)
Sex [*n* (%)]			
Male	35 (58)	40 (66)	10 (67)
Female	25 (42)	21 (34)	5 (33)
Hematologic disease [*n* (%)]			
Myeloid	42 (70)	45 (74)	8 (53)
Lymphoid and lymphoproliferative	16 (27)	14 (23)	6 (40)
Other	2 (3)	2 (3)	1 (7)
Source of HSC [*n* (%)]			
Bone marrow	9 (15)	7 (11)	2 (13)
Peripheral blood	48 (80)	52 (85)	13 (87)
Cord blood	3 (5)	2 (3)	0 (0)
HLA match [*n* (%)]			
HLA-matched identical sibling	17 (28)	13 (21)	3 (20)
Other HLA compatibilities	43 (72)	48 (79)	12 (80)
Conditioning protocol [*n* (%)]			
Myeloablative	30 (50)	26 (43)	8 (53)
Reduced intensity	30 (50)	35 (57)	7 (47)
GVHD prophylaxis [*n* (%)]			
With cyclophosphamide	13 (22)	13 (21)	1 (7)
Without cyclophosphamide	47 (78)	48 (79)	14 (93)

With the exception of age, the results are presented in the format “number of patients (percentage)”.

**Table 2:** Distribution of the blood types of the donor/recipient pair according to the type of event investigated.

	Patients likely to change immune response (*n* (%)):			
	Disappearance A	Disappearance B	Appearance A	Appearance B
Donor/Recipient				
A/B	7 (14%)	N.A.	N.A.	7 (31.8%)
A/O	40 (80%)	N.A.	N.A.	N.A.
AB/A	N.A.	6 (20.1%)	N.A.	N.A.
AB/O	3 (6%)	3 (10.3%)	N.A.	N.A.
B/A	N.A.	8 (27.6%)	8 (14.3%)	N.A.
B/O	N.A.	12 (41.4%)	0 (0%)	N.A.
B/AB	N.A.	N.A.	3 (5.4%)	N.A.
O/A	N.A.	N.A.	43 (76.8%)	N.A.
O/AB	N.A.	N.A.	2 (3.6%)	2 (9.1%)
A/AB	N.A.	N.A.	N.A.	4 (18.2%)
O/B	N.A.	N.A.	N.A.	9 (40.9%)

*N.A.* not applicable.

### Disappearance and appearance of isoagglutinins

Isoagglutinin A disappearance was observed in 82.0% (41/50) of patients for whom this event was expected within one year after allogeneic HSCT, with a median time of disappearance at 38.4 days [IC 95%: 13.5–87.0 days], and a 75th percentile time at 138.6 days [38.4–NA]. Isoagglutinin B disappearance was observed in 96.4% (27/28) of patients, with a median time of disappearance of 15.9 days [10.5–29.1 days], and a 75th percentile time at 29.1 days [12.6–NA]. No reappearance was observed over the one-year follow-up period.

There was no appearance of isoagglutinin B (0/22), whereas appearance of isoagglutinin A was observed in 10.7% (6/56) of patients, with a median (and range) time of appearance of 13.0 [12–20] days for the patients concerned. No appearance was observed among patients after a bidirectional HSCT.

**Fig. 1:**
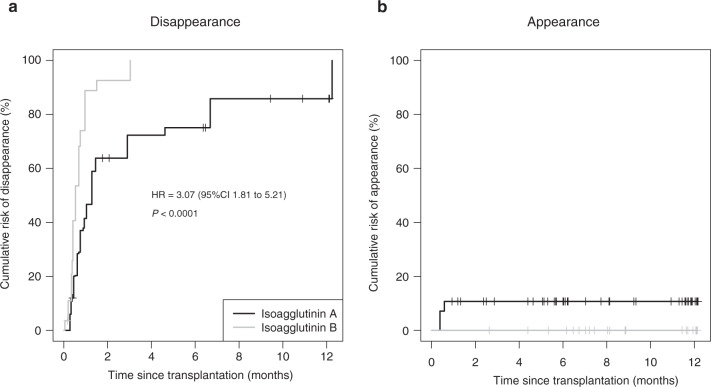
Incidence of disappearance and appearance of isoagglutinins A and B. Cumulative incidence curves for disappearance (**a**) and appearance (**b**) of isoagglutinins A and B. Small vertical lines represent loss to follow-up or death. HR = hazard ratio.

These disappearance and appearance results are presented as cumulative incidence curves in Fig. 1, and the censoring data are detailed in Appendix 3.

### Comparison of the disappearance of isoagglutinins A and B

Among the 50 patients for the event “isoagglutinin A disappearance” and the 28 patients for the event “isoagglutinin B disappearance”, 3 are in common (AB-type donor to O-type recipient). In the analysis comparing the disappearance of isoagglutinins A and B, there are therefore in reality 75 distinct patients, but we considered that the sample was composed of 78 distinct patients.

A significant difference (HR = 3.07; *p* < 0.0001) between the cumulative incidence curves was demonstrated concerning the disappearance of isoagglutinins A and B (Fig. 1).

### Disappearance of isoagglutinin A according to the type of hematological disease

Among the 50 patients in whom disappearance of isoagglutinin A is likely, 66.0% (33/50) had a myeloid diseases and 34.0% (17/50) had another hematological diseases (lymphoid, lymphoproliferative, other).

**Fig. 2:**
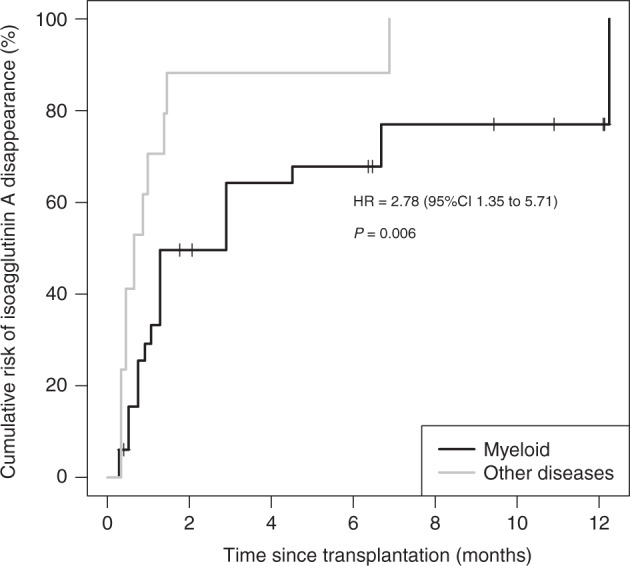
Incidence of isoagglutinin A disappearance according to hematological disease. Cumulative incidence curves of isoagglutinin A disappearance in patients with and without myeloid diseases. Small vertical lines represent loss to follow-up or death. HR = hazard ratio.

A disappearance of these isoagglutinins was observed in 72.7% (24/33) of patients with myeloid diseases, and in 100.0% (17/17) of patients with other hematological diseases. The cumulative incidence curves are presented in Fig. 2, and are significantly different (HR = 2.78; *p* = 0.006). Adjusted to HLA-compatibility, hazard ratio was similar (HR = 3.05; *p* = 0.009). The censored data are detailed in Appendix 4.

This analysis was not performed for isoagglutinin B because of the sample size, which would have led to a similar but less powerful result.

### Disappearance of isoagglutinin A according to the type of HLA compatibility

Among the 50 concerned patients, 24.0% (12/50) had a HLA-matched related HSCT and 76.0% (38/50) had another type of HLA compatibility (HLA-matched unrelated or HLA-mismatched related or not).

A disappearance of these isoagglutinins was observed in 75.0% (9/12) of patients with HLA-matched related HSCT, and in 84.2% (32/38) of patients with other types of HLA compatibility. The cumulative incidence curves are not significantly different (HR = 1.55; *p* = 0.36). Adjusted to hematological disease, hazard ratio was similar (HR = 1.86; *p* = 0.19). The censored data are detailed in Appendix 5.

### Disappearance of isoagglutinin A according to transplantation strategy

There was no significant difference according to intensity of conditioning (HR = 0.87; *p* = 0.70), administration of ATG (HR = 1.24; *p* = 0.56), stem cell source (HR = 1.30; *p* = 0.52), T-cell depletion of the graft (HR = 0.54; *p* = 0.26), PRCA (HR = 0.22; *p* = 0.52) and one-year relapse (HR = 0.51; *p* = 0.19).

## Discussion

Our study includes 42% of ABO incompatibility, which is in agreement with the literature mentioning an ABO incompatibility rate of 30 to 50% in allogeneic HSCT [9]. The higher level of related HSCT explains a lower rate than expected [8, 19].

While the disappearance of isoagglutinins A and B in case of major or bidirectional incompatibility is frequent during the first year after allogeneic HSCT, respectively in 82% and 96% of cases, the appearance of minor or bidirectional incompatibility is much less frequent (11% for isoagglutinin A, or even non-existent for isoagglutinin B in one year). These rates are comparable with the results of Lee et al. [14]. This study shows a disappearance of isoagglutinins (A and B) in 97% of the 40 patients and an appearance in 18% of the 36 patients. The main explanation for the persistence of isoagglutinins is the resistance of plasma cells to conditioning. Studying leukocyte subpopulation chimerism among 12 patients receiving attenuated conditioning, Griffith et al. highlighted this phenomena [20]. Lee et al. also found that HLA incompatibility had a significant impact on isoagglutinins disappearance suggesting a graft versus plasma cell effect [14]. On the other hand, the low rate of de novo appearance of isoagglutinins can be explained by post-allogeneic HSCT immunosuppressive therapy [13]. Another hypothesis could also be related to the presence of ABO antigens on a wide variety of human tissues, which would limit the detection of corresponding antibodies [21].

Time to disappearance for isoagglutinin A was 38 days and 139 for the median and 75th percentile, respectively. These same values are 16 and 29 days for isoagglutinin B, respectively. These values can help the clinician in the follow-up after ABO incompatible allogeneic HSCT, in particular because of the link between the time of disappearance of isoagglutinins and the graft uptake in the erythroid compartment, as highlighted in the study of Lee et al. [22]. However, the median disappearance time of isoagglutinins A and B in 27 patients were respectively 160 and 51 days, which is longer than the time observed in our center. In the 2003 study, Lee et al. also observed a higher median disappearance time of isoagglutinins (without distinguishing between A and B) of 89 days [14]. But in this study with little methodological information, especially about the time between isoagglutinins tests, a lower frequency of control would have led to a late diagnosis. Interestingly, the source of HSC was also inversed between our study and the one published by Lee et al. in 2003. The collection rate of peripheral blood was inferior to 20% in the Korean cohort and superior to 80% in ours, which is more in line with current practices [23]. Given that peripheral stem cell collection are richer in T lymphocytes, it could explain the more precocious disappearance of isoagglutinins in our study. Finally, our study also included a larger patient sample size (75 versus 40).

In another less common situation, these key values of isoagglutinins disappearance could also be clinically relevant to suspect a relapse, in case of a recipient isoagglutinins detection, more than twelve months after a major ABO-incompatible HSCT [11].

In our study, we observed a significantly shorter median time to disappearance of isoagglutinin B than isoagglutinin A (15.9 days vs. 38.4 days) (Fig. 1), which is in agreement with the literature [22, 24]. This difference of disappearance time could be explained by the higher titers of isoagglutinin A than B, commonly observed in immunohematology laboratories.

In a subgroup analysis, a significantly faster disappearance of isoagglutinin A was demonstrated after HSCT following a non-myeloid hematological disease (lymphoproliferative, lymphoid or others), compared to those with a myeloid disease (HR = 2.78). Patients with lymphocytic lineage diseases representing 87.8% of the 41 patients in our study with non-myeloid diseases. Our hypothesis for this difference is in relationship with the main disease which affect lymphocytes. In this setting, lymphocytes function is impaired by the disease and the targeted therapy [25].

As Lee et al. in 2003 reported a significant difference in the isoagglutinins disappearance between HLA-sibling transplantation and the others, we carried out this subgroup analysis [14]. Our study showed a neither rapid nor significant difference between these two groups concerning isoagglutinins A. However, our study has a smaller sample size (only 12 patients with HLA-matched sibling HSCT), which lead to a lack of statistical power.

The median appearance of isoagglutinin A was 13 days for the 6 patients in whom this event occurred. This appearance of isoagglutinins was not associated with the complete entity of a passenger lymphocyte syndrome. Hemolysis was prevented by our local policy of transfusion: transfusions are always ABO-compatible with the recipient and the donor from the day of transplantation. This observation shows that, even if our policy could stress the blood product stock, the preventive administration of group O erythrocyte concentrates is justified [26]. Finally, this de novo appearance of isoagglutinins is a clinically interesting sign of an activated immunity, emanating obligatorily from the donor lymphocytes. Less reliably, Lee et al. in 2003 described a transient decrease followed by an increase around the second month of isoagglutinins titer in the setting of isogroup HSCT [14].

Our study has some limitations, such as the sample size. Thus some interesting subgroups analyses could provide significant results. However, this is, to our knowledge, the first study involving such a large cohort of patients. A second limitation was the short follow-up, particularly for the appearance of isoagglutinins. However, these events seems to appear early. A longer follow-up would also have been interesting to study all the disappearances of isoagglutinin. Third, the analyses frequency was variable between patients and possibly insufficient. Consequently, data could be slightly inhomogeneous and some events could have been detected too late. Finally, although our data are censored following the death of patients during the follow-up period, this is however a minor limitation due to the low number of patients in this case (e.g. *n* = 5 for disappearance of isoagglutinin A).

In conclusion, this study of 136 ABO-incompatible HSCT describes the evolution of isoagglutinins in the year following transplantation and provides ready-to-use values for the physicians. Within one year following HSCT, a vast majority of incompatible isoagglutinins disappears whereas a small minority appears. These findings are clinically relevant given the known relationship between isoagglutinins and preventable immunohematological complications.

## Supplementary information

Appendix legends

Appendix 1

Appendix 2

Appendix 3

Appendix 4

Appendix 5

## Data Availability

All data can be obtained by email request to the corresponding author.
