# Supplementary material for: Kinetics of disappearance and appearance of isoagglutinins A and B after ABO-incompatible hematopoietic stem cell transplantation
Source: Bone Marrow Transplant. 2022 Jun 25;57(9):1405–10. doi: 10.1038/s41409-022-01737-z (PMC9439946; doi:10.1038/s41409-022-01737-z)
Supplement: Supplementary file 1 — Appendix legends [file 41409_2022_1737_MOESM1_ESM.docx]

**Appendix legends**

Appendix 1: Most used conditioning regimens according their categories

O: reduced intensity or non myeloablative (others); M : myeloablative; TBI: total body irradiation; Gy: grays; Bu: Busulfan; CY: cyclophosphamide; Flu: Fludarabine; Treo: treosulfan; Mel: melphalan; ATG : anti-thymocyte globulin, Thio: thiotepa; Cla: cladribine; AMSA: amsacrine

Appendix 2: Distribution of hematological diseases according to the type of event investigated

Appendix 3: Details on death-censored data for disappearance and appearance of isoagglutinins A and B

* : deaths occurred at 100, 152, 167, 168, 328, 341 and 354 days in n=7 patients with disappearance A and at 13, 66, 72, 205 and 335 days in n=5 patients without disappearance A

** : deaths occurred at 86, 178, 207, 246, 259, 269, 337 and 354 days in n=8 patients with disappearance B and at 8 days in n=1 patient without disappearance B

*** : deaths occurred at 41, 86, 131, 153, 166, 168, 169, 178, 183, 190, 207, 214, 246, 335, 337 and 342 in n=16 patients without A disappearance

**** : deaths occur at 168, 205, 214, 248, 253, and 287 days in n=6 patients with no B appearance

Appendix 4: Details on death-censored data for isoagglutinin A disappearance according to the hematological disease

* : deaths occurred at 100, 152, 167 and 341 days in n=4 patients with A disappearance and at 13, 66, 72, 205 and 335 days in n=5 patients without A disappearance

** : deaths occurred at 168, 328 and 354 days in the n=3 patients with disappearance A

Appendix 5: Details on death-censored data for isoagglutinin A disappearance by type of HLA compatibility

* : deaths occurred at 354 days in n=1 patient with A disappearance and at 335 days in n=1 patient without A disappearance

** : deaths occurred at 100, 152, 167, 168, 328 and 341 days in the n=6 patients with disappearance A and at 13, 66, 72 and 205 days in n=4 patients without A disappearance
